# Perceptions of air pollution and health communication for people with asthma among Australia’s Arabic-speaking communities

**DOI:** 10.1093/heapro/daaf113

**Published:** 2025-08-01

**Authors:** Karima Laachir, Nigel Goodman, Bandana Saini, Mustapha Taibi, Penelope J Jones, Sotiris Vardoulakis

**Affiliations:** Centre for Arab and Islamic Studies, College of Arts and Social Sciences, Australian National University, 127 Ellery Crescent, Canberra, ACT 2601, Australia; HEAL Global Research Centre, Health Research Institute, University of Canberra, 11 Kirinari Street, Bruce, ACT 2617, Australia; Sydney Pharmacy School, Faculty of Medicine and Health, University of Sydney, A15 Science Road, Camperdown, NSW 2050, Australia; Woolcock Institute of Medical Research, 2 Innovation Road, Macquarie Park, NSW 2113, Australia; School of Humanities and Communication Arts, Western Sydney University, Locked Bag 1797, Penrith, NSW 2751, Australia; Menzies Institute for Medical Research, University of Tasmania, 17 Liverpool Street, Hobart, TAS 7000, Australia; HEAL Global Research Centre, Health Research Institute, University of Canberra, 11 Kirinari Street, Bruce, ACT 2617, Australia

**Keywords:** culturally and linguistically diverse communities, air quality, asthma, environmental health literacy, health promotion, communication

## Abstract

Air pollution is a major public health risk factor globally and a significant threat to people with respiratory conditions. People with asthma, and particularly those from culturally and linguistically diverse (CALD) backgrounds, are disproportionally affected and have less capacity to protect themselves from air pollution. There is a critical lack of accessible resources and advice for people with asthma who are from CALD backgrounds. This qualitative study aimed to better understand Arabic-speaking Australians’ perceptions of air quality, support their health literacy, and co-design resources to help them reduce their exposure to air pollution. A virtual roundtable discussion was conducted with key stakeholders from Australian Arabic-speaking communities to explore perceptions of air pollution and effective ways to communicate related public health messages to people with asthma within these communities. Australian Arabic-speaking communities generally have low awareness of air pollution. Although they use social media platforms and traditional media widely, more needs to be done to raise their awareness of air pollution and related health issues through targeted bilingual (English-Arabic) messaging and audiovisual material. The importance of religious and other community leaders in promoting environmental and public health messages within the diverse Arabic-speaking communities was highlighted. Future asthma-awareness and air pollution literacy campaigns should be designed in ways that reach CALD communities that have previously been underserved by public health promotion. Culturally sensitive health communication approaches are particularly important as Australia's population continues to diversify.

Contribution to Health Promotion

- People with asthma from culturally and linguistically diverse (CALD) communities are disproportionally affected by air pollution.
- Language and cultural barriers and socioeconomic marginalization pose challenges for promoting air pollution and health messages in CALD communities.
- Perceptions on air pollution in Arabic-speaking communities in Australia were explored to inform the development of targeted promotional materials for improving their environmental health literacy and asthma management.
- Measures aiming to enhance health literacy and improve asthma management in CALD communities need to be culturally concordant with their beliefs, expectations, and experiences.
- Culturally sensitive health communication approaches are important as Australia's population continues to diversify.

## INTRODUCTION

Air pollution is a major public health risk factor with around seven million premature deaths associated with ambient and household air pollution globally every year (WHO 2025). In Australia, exposure to outdoor air pollution from human-made sources, measured as fine particulate matter (PM_2.5_), is associated with ∼2600–3200 deaths every year (AIHW 2021, Hanigan *et al*. 2021), corresponding to an average annual economic burden of over AU$6 billion. There is significant variation in air pollution exposure levels depending on the location and season. During colder months, wood heaters are the dominant source of air pollution in many Australian cities (Borchers-Arriagada *et al*. 2024, Vardoulakis *et al*. 2024). During warmer months, high air pollution events in Australia are typically linked to bushfires and dust storms (Hertzog *et al*. 2024). In the 2019–20 Black Summer, around 10 million Australians experienced unprecedented levels of exposure to bushfire smoke (Vardoulakis *et al*. 2020), resulting in 1305 (95% CI, 705–1908) presentations to emergency departments with asthma (Borchers Arriagada *et al*. 2020). There was also a significant increase in rates of dispensing for all respiratory medications (AIHW 2020, Etherington *et al*. 2025).

Persistant and in cases extreme air pollution caused by bushfires highlight the need for clearer, more accessible, practical, and better-targeted air quality and health advice (Royal Commission 2020, Heaney *et al*. 2021). Despite Australia's high asthma prevalence (∼11% of total population) and increasing population diversity, asthma management and air pollution protection measures amongst culturally and linguistically diverse (CALD) population groups have not been well-studied (Walsh *et al*. 2024).

Current evidence suggests that people with asthma, and particularly those from CALD backgrounds, are disproportionally affected and have less capacity to protect themselves from air pollution (Ramírez *et al*. 2019, Asthma Australia 2024). Over one quarter of Australia's population has a CALD background. In 2021, 27.6% of the Australian population were born overseas and 22.8% Australians spoke a language other than English at home (ABS 2022). Arabic is the second most frequently spoken language other than English at home in Australia. In the 2021 Census, 367 159 people (1.44% of the Australian population) spoke Arabic at home, an increase of around 45 500 on the 2016 Census (ABS 2021).

Despite the availability of health coverage (e.g. subsidized access to general practitioners and listed medicines), CALD communities are often reluctant to use them due to cultural differences, experiences of stigma and discrimination, perceived or actual racism, and barriers to communication (Manderson and Allotey 2003, Khatri and Assefa 2022). This has led to poorer health outcomes in CALD communities in Australia (Henderson and Kendall 2011). Other factors, such as poorly targeted health messaging, lower levels of health literacy, and higher rates of exposure to the social and environmental determinants of ill health, may have also contributed to health outcome disparities (AIHW 2024).

General practitioners perceive that treating CALD patients with asthma is difficult and that there are multiple barriers that impact treatment, including issues of autonomy, language, accessibility and engagement, health literacy, and cultural beliefs (Alzayer *et al*. 2019). A study carried out in Melbourne and Sydney showed that language and, to a lesser extent, cultural barriers were regularly experienced by pharmacists managing CALD patients with asthma (Alzayer *et al*. 2021). Research suggests that lower health literacy, cultural beliefs and language barriers, alongside limited knowledge about asthma and air pollution exposure, are affecting asthma control in the Arabic-speaking population in Australia (Alzayer *et al*. 2018).

There is therefore a critical need for accessible information and education on air pollution and on strategies to manage exposure and respiratory health for CALD population groups. This is particularly urgent for population groups that may be at a higher risk of developing adverse health outcomes because they are socio-economically marginalized or overlooked by current health messaging due to language barriers and a lack of trust in the systems or individuals conveying these messages. The present study aimed to explore perceptions of air pollution and effective ways to communicate related public health messages to Australia's Arabic-speaking communities, and particularly to people with asthma within these communities.

## MATERIALS AND METHODS

### Community translation

We used community translation as an overarching framework to address the issue within the Arabic-speaking Australian community (Al-Juhaishi *et al*. 2024). Community translation is a language service empowering minority language groups for appropriate action by ensuring access to public service information, including multilingual health promotion materials (Taibi 2023). Effective community translation takes into consideration the sociolinguistic context of the target community, their literacy levels, and their communication preferences (Taibi 2018). The community translation framework requires consultation with and involvement of CALD stakeholders, which are key to empowerment and improved health literacy and outcomes in these communities (Taibi *et al*. 2019). As part of this consultative process, we employed an exploratory roundtable discussion methodology, which is a common method of engagement in community-based participatory health promotion research (Bammann *et al*. 2021).

### Data collection and analysis

A virtual roundtable discussion was conducted with key stakeholders from Arabic-speaking communities and service providers who worked with these communities and had experience in devising strategies for disseminating health messages within their communities. The discussion was moderated with the help of a topic guide focused on the study aim to allow coverage of key issues, including:

- Arabic-speaking Australians’ perceptions of air quality and the air pollution literacy of health professionals who treat people with asthma.
- The design of health promotion resources to help this specific community reduce air pollution exposure, particularly in relation to:
  - barriers and enablers and cultural aspects that need to be taken into consideration in public health strategies aiming to reduce exposure to outdoor air pollution;
  - requirements and considerations for developing resources to improve the air pollution literacy of people with asthma and of their caregivers in Arabic-speaking communities;
  - priorities and gaps that need to be addressed through policy action to improve air quality and the health and well-being of people with asthma.

These issues were explored by asking a series of questions about participants’ experiences of air pollution (e.g. from bushfires, wood heaters, road traffic) in their communities, its impacts on health outcomes (e.g. asthma), and related preventive measures. To keep the discussion as natural as possible, research team members led specific discussion items in a conversational style. Facilitation techniques that included probing, conversation steerage, and ensuring opportunities to share ideas were provided to all participants.

The roundtable discussion was fully recorded and transcribed for the purposes of accurate derivation of core themes. Careful reading of the transcribed notes by the research team was followed by an in-depth discussion of nuanced and subliminal messages. The final step in the analysis was the categorization of data-derived ideas into relevant themes.

### Participant recruitment

Participants in this roundtable included Arabic-speaking community organizations, as well as environmental public health, cultural and linguistic experts from three Australian jurisdictions. Purposive sampling was used to recruit roundtable participants. They were recruited through organizations that work directly with Arabic-speaking communities in Australia. Ten stakeholders from six community organizations from New South Wales (NSW), Victoria, and the Australian Capital Territory (ACT), and six academics participated in the roundtable.

We anticipated that stakeholder perspectives may differ across the community, health, and linguistic domains represented at the roundtable. Capturing these diverse perspectives would ensure that responses covered a range of views on this issue. Identifying core themes within the responses enabled us to map out key considerations in developing targeted air pollution and health communication resources for Arabic-speaking communities.

Details of the recruitment process, participation, and roundtable questions are provided as Supplementary material.

## RESULTS

The results of our analysis are presented under the three core themes identified within the responses to the roundtable questions and summarized in Table 1.

**Table 1.** Key findings from the roundtable discussion on air pollution and health communication among Australia's Arabic-speaking communities.

Current perceptions	Low community awareness of outdoor air pollution as a health issue
	Active and passive smoking of tobacco and shisha perceived as the main air quality–related health risk factor
	Diversity of Arabic-speaking community requires nuanced communication approaches
	Socio-economic factors potentially interfering with environmental health literacy
	Cultural and religious factors potentially interfering with environmental health literacy
Recommended communication approaches	Face-to-face engagement, in addition to communication via diverse media channels
	Social media and radio campaigns targeted to Arabic-speaking communities
	Culturally sensitive audiovisual communication of air pollution and health messages (e.g. podcasts, videos, infographics)
	Bilingual (English-Arabic) and Arabic-specific communication in standard Arabic
	Brief, clear messaging (e.g. factsheets) likely to be more effective than longer documents
	Role for religious leaders and institutions in promoting public health messages
	Bilingual health workers (e.g. pharmacists) can play an important role in promoting air pollution and health literacy
	Community workshops in Arabic, combining food and socializing for families, to raise awareness and share ideas

### Theme 1: Arabic-speaking community's awareness of the health impacts of air pollution

Participants were asked about their community's experience of air pollution and the impacts on health, with a specific question ‘How well prepared do you think Arab-Australian communities are to deal with this problem?’ It was noted that there is low awareness in Arabic-speaking communities, with a more pressing focus on daily needs and survival than air pollution.

A stakeholder highlighted the importance of a ‘unified language … for air pollution awareness’ and provided an example of a school dealing with playground dust affecting students with asthma. Another participant suggested that traffic is seen as a major pollution source, with a need for government to support electric vehicle adoption, while also noting the role of ‘passive smoke’ from cigarettes and, in the community context, ‘shisha’ (i.e. waterpipe for smoking tobacco). A number of participants raised the issue of smoking and suggested that the government should educate in simple language and involve new arrivals in awareness programs.

Another participant stated that ‘Different cultures prepare for disasters differently; communication needs to be culturally sensitive’, but also that the ‘Arab community is diverse; awareness campaigns should respect cultural differences and focus on individual needs’.

### Theme 2: community preparedness for managing air pollution impacts

Participants were asked when there is an episode of air pollution, how people with asthma in their communities manage the condition. They noted a lack of awareness and preparedness, partly as a consequence of inadequate language-specific resources. Participants specifically noted socio-economic, linguistic, and cultural barriers to preparedness, including a lack of public health resources related to air pollution and asthma in Arabic language. A participant noted that ‘Asthmatics use inhalers but lack broader preparation’.

Suggestions for improvement of the preparedness of people with asthma in their community included the use of religious centres for communication, use of Arabic community radio as well as social media, developing culturally sensitive communication resources (in Arabic and English), and focusing on key groups (e.g. mothers, children).

### Theme 3: preferences for communications about managing air quality and health impact

Participants were asked which areas of air quality and health communication may be more effective, and what they feel is most important for people in the community to know about, or understand, in relation to air pollution and health. Socioeconomic barriers to accessing quality educational materials were raised as an important issue. For example, it was highlighted that those materials ‘…often don't reach low socio-economic groups’.

Several stakeholders noted the need for effective communication through traditional media (e.g. radio), social media (including visual aids), and face-to-face engagement (e.g. workshops) to improve understanding of the sources of air pollution and environmental risk factors that people in the community are exposed to.

Participants were further asked about practical suggestions on how to consult community members when designing targeted communication tools and health messaging for Arabic-speaking communities. They noted existing channels that can be utilized including translators and interpreters, community leaders, and bilingual health workers (e.g. pharmacists). They also expressed the need to cover the diversity of the Arabic-speaking community in the consultation process. A participant highlighted that ‘awareness campaigns should respect cultural differences and focus on individual needs’.

## DISCUSSION

Australians of Arab descent belong to diverse ethnic and religious groups united by shared cultural and linguistic heritage. The Arabic-speaking community in Australia has low awareness of air pollution and related health effects for people with asthma or other chronic health conditions. They also feel less connected with asthma care pathways than other Australians, have varied health beliefs not considered by conventional healthcare streams, and are less able to self-manage their (or their child's) asthma (Alzayer *et al*. 2018, Al-Juhaishi *et al*. 2024). Therefore, there is a need for clear, concise, and targeted health information to support the community in better managing asthma and reducing exposure to environmental hazards. In particular, there is a need to increase awareness on how to reduce personal, household, and community exposure to air pollution that can trigger or exacerbate asthma symptoms (Goodman *et al*. 2025), in parallel with additional support to promote asthma awareness. Communication tools should be co-designed with health service providers, consumer peak bodies, and Arabic community organizations. The dissemination and uptake of these tools could be enhanced through indirect promotion by Arabic-speaking religious, cultural, and educational centres and the involvement of bilingual health professionals (Singh *et al*. 2024).

In terms of language usage, standard Arabic (rather than spoken Arabic dialects) should be prioritized, with audio messaging delivered via community radio, which was noted as an effective medium that reaches different age groups (i.e. older and younger people) in the Arabic-speaking community. Various social media platforms (e.g. WhatsApp, Facebook) and visual information (videos, infographics) could be used to further engage the community.

In addition, noting the diversity of the Arabic-speaking community in Australia, community-based workshops tailored for different groups (depending on age and cultural background) and provided in publicly accessible venues, were noted as a potentially useful way for raising awareness on air pollution and health protection. This may include a role for religious and other community leaders in promoting environmental health literacy in Arabic-speaking communities.

This study provided an opportunity to gain community stakeholder insights in air pollution and health literacy and forge an interdisciplinary collaboration between public health, environmental and social scientists, Asthma Australia, and Arabic organizations. These findings help to increase the understanding and knowledge of health service providers and Australia's Arabic-speaking communities on communication options for raising awareness on air pollution and health protection. A key outcome was the development of simplified English and Arabic language factsheets, infographics, and social media messaging (HEAL 2025).

Further consultation should explore Arabic-speaking community members’ perspectives, including the lived experiences of people with asthma from these communities. This would provide additional insights into barriers and enablers for the development of culturally appropriate and effective air pollution exposure reduction strategies and related public health messaging from a consumer perspective. Similar collaborative research projects focusing on other CALD communities that may be at higher risk from exposure to air pollution and other environmental hazards would be beneficial.

## CONCLUSION

There is limited awareness of the adverse effects of air pollution on people with asthma in Arabic-speaking communities in Australia. This study informed the development of simplified English and Arabic language resources for promoting interventions to reduce air pollution exposure and improve health outcomes in Arabic-speaking communities. Targeted measures aiming to enhance health literacy and improve asthma management in CALD communities need to be culturally concordant with the beliefs, expectations, and experiences of such population groups.

Culturally sensitive health communication approaches are particularly important as Australia's population continues to diversify, and more people are impacted by air pollution and extreme events such as bushfires. Nuanced health messaging, developed in consultation with CALD communities, and with an understanding of the specific needs and barriers they face, will ensure that health promotion campaigns equitably reach all Australians.

## Supplementary Material

daaf113_Supplementary_Data

## Data Availability

The data underlying this article cannot be shared publicly due to the privacy of individuals who participated in the study.
