# Supplementary material for: Perceptions of air pollution and health communication for people with asthma among Australia’s Arabic-speaking communities
Source: Health Promot Int. 2025 Aug 1;40(4):daaf113. doi: 10.1093/heapro/daaf113 (PMC12314265; doi:10.1093/heapro/daaf113)
Supplement: daaf113_Supplementary_Data [file daaf113_supplementary_data.docx]

**Perceptions of air pollution and health communication for people with asthma among Australia’s Arabic-speaking communities**

**Supplementary Information**

**1. Participants recruitment**

Purposive sampling was used to recruit roundtable participants. Participants were recruited through organisations that work with the priority populations, through personal contacts, and professional networks. Several team members worked with Arab community organisations in Australia and had established working relationships with service providers or stakeholders. These contacts were invited to participate in the roundtable. A maximum of 20 stakeholders representing community, research, and health organisations were invited. Ten stakeholders from six community organisations (Table S1) and six academics accepted the invitation.

As the roundtable was intended to be a scoping exercise that would inform future research activities, a priori sample size calculations were not performed.

Table S1. Community organisation participation in roundtable discussion

| **Affiliation** | **State or Territory** |
| --- | --- |
| Women’s Health Matters Organisation and Health in my Language Program | Australian Capital Territory |
| Arab/Australian Communities in Canberra | Australian Capital Territory |
| Arab Council Australia | New South Wales |
| Australian Council of Women Affairs; and Al-Nawawi Centre | New South Wales |
| Arabic Interpreters and Translators Australia | New South Wales |
| Victorian Arabic Speaking Services network | Victoria |

**2. Roundtable questions**

1. *Please tell us about your experiences of air pollution (e.g. from bushfires, wood heater smoke, traffic pollution) in your community and how it impacts health (e.g. asthma). How well do you think are Arab-Australian communities prepared to deal with this problem?*
2. *Which areas of air quality and health communication matter the most to you? What do you feel it is most important for people in the community to know about, or understand, when it comes to air pollution and health?*
3. *When there is an episode of air pollution, how do you feel those with asthma in your communities manage the condition?*
4. *Do you have any ideas about how the preparedness of those with asthma in your community can be improved? E.g. access to information (language), cultural proficiency of health professionals, culturally appropriate self-management tools (action plans, apps), community engagement and discussion at key community venues or by prominent community members?*
5. *Do you have practical suggestions about how to consult community members when designing targeted communication tools and health messaging for Arabic-speaking communities?*
6. *What adaptations would air pollution and/or health messaging to Arabic-speaking community members require?*
